# A novel emergency department based prevention intervention program for people living with HIV: evaluation of early experiences

**DOI:** 10.1186/1472-6963-7-164

**Published:** 2007-10-15

**Authors:** Michael S Lyons, Dana L Raab, Christopher J Lindsell, Alexander T Trott, Carl J Fichtenbaum

**Affiliations:** 1 Department of Emergency Medicine, University of Cincinnati College of Medicine, Cincinnati, OH, USA; 2 Division of Infectious Diseases, University of Cincinnati College of Medicine, Cincinnati, OH, USA

## Abstract

**Background:**

HIV prevention is increasingly focused on people living with HIV (PLWH) and the role of healthcare settings in prevention. Emergency Departments (EDs) frequently care for PLWH, but do not typically endorse a prevention mission. We conducted a pilot exploratory evaluation of the first reported ED program to address the prevention needs of PLWH.

**Methods:**

This retrospective observational cohort evaluation reviewed program records to describe the first six months of participants and programmatic operation. Trained counselors provided a risk assessment and counseling intervention combined with three linkage interventions: i) linkage to health care, ii) linkage to case management, and iii) linkage to partner counseling and referral.

**Results:**

Of 81 self-identified PLWH who were approached, 55 initially agreed to participate. Of those completing risk assessment, 17/53 (32%, 95 CI 20% to 46%) reported unprotected anal/vaginal intercourse or needle sharing in the past six months with a partner presumed to be HIV negative. Counseling was provided to 52/53 (98%). For those requesting services, 11/15 (73%) were linked to healthcare, 4/23 (17%) were coordinated with case management, and 1/4 (25%) completed partner counseling and referral.

**Conclusion:**

Given base resources of trained counselors, it was feasible to implement a program to address the prevention needs for persons living with HIV in an urban ED. ED patients with HIV often have unmet needs which might be addressed by improved linkage with existing community resources. Healthcare and prevention barriers for PLWH may be attenuated if EDs were to incorporate CDC recommended prevention measures for healthcare providers.

## Background

Many people living with HIV (PLWH) continue to engage in high risk behavior [1-8], and transmission by PLWH who have been exposed to drug regimens accelerates drug resistance[9,10]. The public health community is therefore emphasizing the critical importance of prevention interventions among PLWH [11-16]. The CDC has specifically recommended integration of HIV prevention interventions into the clinical care of HIV-infected patients, and the efficacy of provider delivered behavioral interventions is increasingly demonstrated[16,17]. Clinicians have been called on to screen for high-risk behaviors, to communicate and serially reinforce prevention messages, to refer patients for services such as substance abuse treatment, and to facilitate partner counseling and referral services [14]. Despite this, many physicians do not yet focus on prevention for PLWH[18,19]. With an expanding evidence base and promotion by public health authorities, clinician attention to prevention for PLWH may increase, but such progress could only affect those PLWH who are in a relationship with a care provider.

Emergency departments (EDs) routinely receive PLWH in a medical setting where an opportunity for interaction exists. EDs account for over 110 million visits annually[20], including people from every background, socioeconomic group, and health status [21-23]. Because EDs are a key component of the health care safety net, patients not linked with health services are particularly likely to seek care in an ED when medical concerns arise [24-27]. Patients accept preventive services and value counseling during ED visits [28-32]. The ED is therefore increasingly recognized as an excellent location for public health programs, despite the novel and controversial nature of this role[24,25,29,31,33-35]. No work has been done, however, to implement prevention interventions for PLWH in the ED setting.

Our ED has incorporated a health-department funded HIV counseling and testing program that has not previously focused on PLWH into clinical practice since 1998[35,36]. With the advent of the CDC Advancing HIV Prevention Initiative[11] and the increasing acceptance of ED-based public health intervention, we have expanded our services to additionally target PLWH for ED prevention interventions. This pilot exploratory evaluation describes how a program to address the prevention needs of PLWH has been implemented in an urban ED. The theoretical framework for the intervention and preliminary outcomes of the first 6 months of operation are discussed.

## Methods

### Study Design

This is a retrospective observational cohort study using review of programmatic and clinical data to describe the early experience with an ED based prevention intervention for PLWH. The study was approved with a waiver of informed consent by the University of Cincinnati Institutional Review Board (IRB # 06-09-07-01).

### Setting

The Prevention for PLWH Program is located in the ED of an urban, teaching hospital that serves as the primary provider of emergency services for the region's indigent population. In 2005 according to US census data, the county in which the hospital is located had an estimated population of 806,652 that was 25.4% black, 71.7% white, and 1.5% Hispanic. In 2003, the number of PLWH in the county was 1,723. The annual ED census is over 85,000 patient visits, with two thirds of patients having Medicaid or no insurance; 57% are black, 39% white, and 0.5% Latino. Almost all patients are over 18 years old; an ED dedicated to the care of children is located several blocks away. Based on 2005 billing databases, the ED provided care for 322 patient visits where the discharge diagnosis included HIV; this estimate does not include visits where: 1) PLWH were seen for non-HIV related reasons and the physician did not include HIV among the written discharge diagnoses, and 2) no patient bill was generated.

### The ED-based HIV Prevention for PLWH Program

The prevention for PLWH program was developed to integrate with our established HIV counseling and testing program, which operates as an adjunct to the clinical activities of the ED and has been described in detail elsewhere[35,36]. Briefly, the program traditionally offered services within the ED only to patients not yet diagnosed with HIV. It is staffed 24 hours a day by dedicated counselors. The prevention for PLWH program is implemented by these counselors with financial support from the state's Department of Health, and the program collaborates closely with the local academic Infectious Disease practice, case management organizations, and the health department.

#### Selection of Participants

All patients seen in the ED who self-identify as a PLWH are eligible for participation; PLWH frequently disclose their status at triage or are identified by physician interview or review of chart records during the routine course of ED care. Patients not referred directly to the program by ED staff may be identified by the counselors who routinely screen ED patient bedside records. Once a patient is identified, he or she is approached and the intervention is described in detail. The patient is told that participation is voluntary and written consent is required. Presence of physical or mental disability of sufficient severity to interfere with the ability to understand and consent for the intervention, as determined by program counselors in consultation with the treating physician, excludes enrollment. Patient consent includes participation in the clinical program and storage of patient information for programmatic quality assurance and quarterly reporting to funding agencies. If patients desire active linkage with any partnering agencies, additional written consent is required to transmit patient information.

#### Interventions

Prevention for PLWH combines a risk-assessment and counseling intervention with three linkage interventions: i) linkage to health care, ii) linkage to case management services, and iii) linkage to partner counseling and referral services. Interventions were selected in accordance with CDC recommendations for incorporating HIV prevention into the medical care of persons living with HIV[14]. The CDC's Advancing HIV Prevention: Interim Technical Guidance for Selected Interventions[37], and the Diffusion of Effective Behavioral Interventions[38] project "Partnership for Health" (PFH)[16,39], were used to guide intervention implementation. The program was designed to mimic as much as possible the PFH model, which is a brief, provider-delivered, counseling program for PLWH. PFH is based on social cognitive theory that uses message framing, repetition and reinforcement to increase the patient's knowledge, skills, and motivations to practice safer sex. The provider and patient identify behavioral goals and the provider gives the patient referrals to any needed services. Application of the PFH model to the emergency care setting required several amendments. The repetition of counseling across a series of encounters is not possible in the ED due to the episodic, unscheduled care environment. Also, we did not hang posters or distribute brochures in the ED waiting room due in part to the small minority of patients to whom these would be relevant.

#### Risk Assessment and Risk Reduction Counseling

The CDC's recommended client centered risk assessment and risk reduction counseling plays a key role in helping patients reduce risky behaviors and maintain safer practices [11,14]. Participants complete a semi-structured, personalized interview and counseling session. The session begins by conducting a risk assessment inventory with individual factors such as age, race, gender, and health status considered. All counselors are trained in appropriate levels of interaction for the patient's culture, language, sex, sexual orientation, age, and developmental status. The risk assessment involves a face-to-face discussion and identifies patients at highest risk of transmitting HIV. This includes patients who engage in sex or drug-injection practices that may lead to transmission, who have a current or recent STD, or who have mentioned other items of concern to the counselor. Specifics such as who (gender and number of partners), what (specific sexual practices, drug-related behaviors), and where (partner meeting venues, venues that promote drug use) are discussed. Once the assessment inventory is complete, the patient's risk factors are evaluated and the counselor offers brief, individualized counseling that focuses on behaviors, circumstances, and behavioral goals[14]. An appropriate risk-reduction plan is discussed and agreed upon. Barriers to behavior change and the skills needed to make the change are explored. Patients receive accurate information regarding factors that influence HIV transmission and methods for reducing transmission risk. Effective methods for preventing transmission to non-infected persons include sexual abstinence, consistent and correct use of condoms, and sex with partners of the same HIV status. Misconceptions about transmission are identified and corrected.

The risk assessment and risk reduction counseling takes between 20 and 40 minutes. Given the length of this intervention component, there are times where interruptions are required to accommodate other aspects of the clinical encounter. In the event patients are ready for discharge before the intervention is completed, counselors are instructed to move patients to an unoccupied area of the ED so that the patient room will be available for the next patient, but we did not assess the frequency of this occurrence.

#### Linkage Interventions

Patients found to need referral services during counseling are enrolled in the referral/linkage component of the program. A similar process of active linkage is used for each of these interventions. With active linkage, the counselor contacts the referral agency on behalf of the patient to schedule visits. To facilitate linkage for visits occurring during nighttime and weekend hours, innovative approaches were developed. First, the patient signs an agency specific consent form specifying the extent to which information can be shared with that agency. The patients are then given two choices: 1) have the agency contact the patient directly, or 2) have the counselor contact the agency on the patient's behalf and set up an appointment. Linkages involving either direct contact from the agency or direct contact from the counselors are completed by telephone. The consent form is faxed to the agency to verify the patient's permission to release information. If the patient does not wish to be actively linked by the counselor, the patient is passively referred, whereby the patient is given the needed information to self-initiate contact with appropriate agencies.

Linkage to health care aims to create a patient-provider relationship, which is of primary importance for HIV prevention. Patients who are in need of a medical provider and sponsored prevention for positives interventions are linked to the on campus Infectious Disease Center. Referrals for issues related to sexual health such as birth control, Pap smears, and drug rehabilitation are offered as a matter of course, but active linkage is not provided.

PLWH are also frequently struggling with psychosocial factors such as mental illness, substance abuse, and homelessness. Case management can address these barriers to effective healthcare and prevention [37,40-42]. Patients who are in need of case management services are linked to a case management program available through local community-based organizations. Case management services include, but are not limited to, support services, educational forums, advocacy, housing assistance, home health care coordination, crisis intervention, and chemical and alcohol dependency programs.

Partner counseling and referral services are a cost-effective means to identify those at highest risk for undiagnosed HIV infection [43-49]; up to 40% of exposed partners may test positive[46]. For partner counseling and referral services, patients are asked to provide partner(s) contact information for the counselor to forward to the local health department. The patient's identity is not shared with the health department unless the patient requests the health department to contact them rather than providing the partner information directly to the counselor.

The time required for linkage interventions is relatively minimal. The risk assessment inventory readily indicates whether the patient is in need of health care, case management, or partner notification services. Linkage to any of these services involves explaining what is offered by the agency, why it would be appropriate for the patient to complete the linkage, and how the consent process works. The process generally takes between 5 and 10 minutes depending on the patient's familiarity with the service and the number of linkages required.

#### Counselor Training

All program staff are trained according to CDC guidelines for client centered HIV prevention counseling [50]. In addition, they received training specific for intervention among PLWH, which consists of a 4-hour didactic session combined with interactive role-playing exercises in intervention delivery. The training session was designed according to 1) guidelines for the PFH intervention and 2) general recommendations for incorporating HIV prevention into the medical care of PLWH. Additionally, procedures for documentation of risk assessment and consent were reviewed. Training was adapted to focus on health care, case management, and partner notification referral services that were available to partner with our program. To reinforce training, counselors were provided with a worksheet outlining intervention procedures, and they were also given the option of shadowing the program coordinator. Subsequent to initial training, the counselors' performance is directly observed for one encounter, and periodically thereafter based on review of completed encounter forms; all forms recording the patient encounter are quality assured with concerns fed back to the counselors for remediation.

### Data Collection and Analysis

The program maintains an ongoing clinical database for quality assurance and reporting functions. This archive includes risk information, details of the counseling provided, and linkage or referral details. The database is cross-referenced with referral agencies quarterly to determine whether the patient attended their scheduled referral visit. Data for this study were extracted from the clinical database. For the purposes of this evaluation, we considered feasibility in the context of resources available and defined it as the ability to enroll patients into the program in the ED setting. The primary programmatic outcome considered was patient linkage; a successful linkage was defined to occur when a patient was initially seen by an agency to which they were referred within 3 months of the index ED visit. Data were stratified by whether or not the patient had an ongoing relationship with a medical provider. This was done to take into account potential differences between patients who were or were not already under the direct care of a medical provider. An ongoing relationship with medical provider was defined as the patient's self-report when asked, "Are you currently being treated medically for HIV or seen regularly by a doctor?" Data are described using means and standard deviations or medians and ranges for continuous variables, and frequencies and proportions for categorical variables. Where appropriate, 95% confidence intervals (95 CI) for proportions were computed using the score method with continuity correction.

## Results

From January 15, 2006 to June 30, 2006, a total of 81 self-reported PLWH were approached in the ED, of whom 55 (68%) initially consented to participate in the program. Mean age of approached patients was 39 years (range 23 years to 55 years); 60% of patients were black and 76% were male (Table 1). Of those agreeing to participate, 43/55 (78%, 95 CI 64% to 88%) claimed to have an ongoing relationship with a medical care provider, 10/55 (18%) claimed not to have an ongoing relationship with a medical care provider, and 2/55 (4%) did not complete risk assessment. Of those who refused to participate in the program, 12/26 (46%, 95 CI 27% to 66%) cited an active relationship with a medical care provider as their reason for refusal. Others were not interested (7/26), unable to consent (2/26), or gave other reasons (4/26) such as living out of state, too much pain, alternative plans, or prior participation. One PLWH declined to give a reason for refusal.

**Table 1:** Characteristics of persons approached for prevention intervention and counseling

		**NON-REFUSERS (N = 55)**		
		
	**REFUSERS (N = 26)**	**Relationship with Medical Care Provider (N = 43)**	**No Relationship with Medical Care Provider (N = 10)**	**Did Not Complete Risk Assessment (N = 2)**
**Age**				
20–29	1 (3.8)	4 (9.3)	1 (10.0)	0 (0.0)
30–39	9 (34.6)	18 (41.9)	6 (60.0)	1 (50.0)
40–49	15 (57.7)	17 (39.5)	2 (20.0)	1 (50.0)
50+	0 (0.0)	4 (9.3)	1 (10.0)	0 (0.0)
Unknown	1 (3.8)	0 (0.0)	0 (0.0)	0 (0.0)

**Race/ethnicity**				
African-American	14 (53.8)	27 (62.8)	8 (80.0)	0 (0.0)
White	9 (34.6)	15 (34.9)	2 (20.0)	2 (100.0)
Other	3 (11.5)	1 (2.3)	0 (0.0)	0 (0.0)

**Sex**				
Male	17 (65.4)	31 (72.1)	8 (80.0)	2 (100.0)
Female	9 (34.6)	12 (27.9)	2 (20.0)	0 (0.0)

**Insurance Status**				
Public Assistance	10 (38.5)	21 (48.8)	3 (30.0)	2 (100.0)
Self	11 (42.3)	22 (51.2)	6 (60.0)	0 (0.0)
Private Insurance	3 (11.5)	0 (0.0)	1 (10.0)	0 (0.0)
Unknown	2 (7.7)	0 (0.0)	0 (0.0)	0 (0.0)

**Risk Assessment^a^**				
MSM		21 (48.8)	3 (30.0)	
IDU		4 (9.3)	3 (30.0)	
MSM/IDU		4 (9.3)	2 (20.0)	
Sex with at-risk partner		7 (16.3)	2 (20.0)	
STD		4 (9.3)	2 (20.0)	
Sex using drugs/alcohol		11 (25.6)	3 (30.0)	
Heterosexual contact only		10 (23.3)	2 (20.0)	
Victim of sexual assault		1 (2.3)	0 (0.0)	
No acknowledged risk		15 (34.8)	4 (40.0)	

**Sexual Activity (last 6 months)^a^**		**N = 24 (55.8%)**	**N = 4 (40.0%)**	
				
*Seroconcordant intercourse*		2 (8.3)	1 (25.0)	
Partner aware of HIV status		2 (8.3)	1 (25.0)	
Always uses condoms		0 (0.0)	0 (0.0)	
Sometimes uses condoms		2 (8.3)	1 (25.0)	
Never uses condoms		0 (0.0)	0 (0.0)	
				
*Serodiscordant intercourse*		22 (91.7)	3 (75.0)	
Partner aware of HIV status		17 (70.8)	1 (25.0)	
Always uses condoms		13 (54.2)	1 (25.0)	
Sometimes uses condoms		5 (20.8)	2 (50.0)	
Never uses condoms		4 (16.7)	0 (0.0)	

Data are presented as frequencies and percentages.

^a ^Data are not available for patients refusing participation and patients not completing risk assessment

Of patients completing risk assessment, 24/53 (45%) reported that they were either married or partnered and 25/53 (47%) reported that they were not sexually active in the past 6 months. For those who reported being sexually active, 21/28 (75%) said their partners knew their HIV status and 25/28 (89%) reported partners who were either HIV negative or HIV status unknown. For patients with serodiscordant partners, 4/25 (16%) reported never using condoms. Sexual activity, partner awareness, and condom use over the last 6 months is reflected in Table 1. Overall, 17/53 (32.1%, 95 CI 20.3% to 46.5%) reported activities placing others at risk for HIV infection, defined as unprotected sexual contact with a serodiscordant partner or needle sharing.

The number of patients accepting each intervention and the number of successful linkages, (active or passive) are shown in Table 2. All but one patient accepted counseling; this patient requested case management linkage. Medical care services were requested by 15/53 (28%) patients. Of those currently claiming to have a relationship with a medical care provider for HIV related illness, 6/7 (86%) were successful in re-establishing contact with an HIV care provider; these patients requested program assistance despite their previous care relationship due to such factors as missed appointments, temporary medication non-compliance, or the need to make an appointment or otherwise resume their care relationship. Of patients not currently being seen by a medical care provider for HIV-related illness, 5/8 (63%) were successful in medical care linkage.

**Table 2:** Interventions offered and completed

Intervention Offered	**Relationship with Medical Care Provider (N = 43)**		**No Relationship with Medical Care Provider (N = 10)**	
	
	Accepted	Completed	Accepted	Completed
Case Management	15 (34.9)	4 (9.3)	8 (80.0)	0 (0.0)
Medical Care	7 (16.3)	6 (13.9)	8 (80.0)	5 (50.0)
Partner Notification	2 (4.7)	1 (2.3)	2 (20.0)	0 (0.0)
Counseling	42 (97.7)	42 (97.7)	10 (100.0)	0 (0.0)

Data available for the 53 patients completing risk assessment and stratified by whether or not they claimed a relationship with a medical care provider. Data are presented as frequencies and percentages.

Case management services were requested by 23/53 (43%) patients. Of those claiming to be in a relationship with a medical care provider, 4/15 (23%) were successful in re-establishing contact with case management services. None of the 8 patients not in an ongoing relationship with a medical care provider followed up with case management services. Assistance with partner notification was requested by 4/53 patients (8%), 2 of whom were already being seen by a medical care provider and 2 who were not. One of the patients already in an ongoing relationship with a medical care provider was successful in establishing a local health department linkage for assistance with partner notification services.

## Discussion

Our pilot exploratory evaluation shows that it is feasible, with appropriately dedicated resources, to enroll PLWH in a program of prevention interventions during an ED encounter. ED-based prevention initiatives are never easily implemented, and the content and scope of this project represents a significant advance in the use of the ED for prevention services. Our theoretical construct and justification for this intervention focused on the following: 1) PLWH who are without sufficient support services are at risk of transmitting HIV, 2) prevention of transmission by PLWH attenuates the spread of drug resistant strains, 3) PLWH without linkages to support services are likely to desire such connections and be able to benefit from them, 4) identification of PLWH in need of support services is not straight forward since agencies dedicated to PLWH are frequently unaware of those they are not serving, and 5) PLWH who do not perceive an availability of other resources are likely to seek support in EDs, particularly when medical concerns arise.

It is important to contextualize our findings; any level of success is notable given the target population and the difficult environment of an urban ED. Psychiatric conditions, substance abuse, and other factors leading to patient disadvantage and ED utilization will also make successful intervention more difficult; our target population, by definition, is difficult to assist. The risk profile of identified patients was, in our experience, limited by patient reluctance to provide accurate information about activities they knew to be potentially harmful or responded in such a way that led the interviewer to question the veracity of the response provided. We note that distrust in medical settings is not uncommon [51-53] and that there may be fear of legal repercussions for the spread of HIV infection[54]. Also, privacy in overcrowded EDs, where patient beds are often placed in hallways, is a significant barrier. Patients in the ED are also sometimes unable or unwilling to participate in non-emergent services because of acute pain, illness, or other circumstances such as frustration or time pressure resulting from treatment delays.

Over time, our program has the potential to expand its contribution beyond single patient encounters and to significantly enhance aggregate understanding of risk behaviors and even transmission patterns among a population of underserved PLWH that is otherwise difficult to identify. For example, even if a patient cannot identify recent partners, broad patterns such as geographic locations and types of social activity are already becoming apparent to our counselors. Our results could ultimately be used to refine estimates of unmet need to assist planning by health authorities and policy makers. We also recognize that even though the ED is an episodic care environment, serial intervention for PLWH who visit the ED repeatedly may be possible, and could lead to additional successes. We have observed that patients are greatly appreciative when the availability and description of various services is explained. Even if linkage with support services is not initially possible, the explanation of social services to patients may prime future attempts at successful contact or enhance the ability of this population of patients to self-advocate for needed services.

To our knowledge there are no prior studies addressing prevention intervention for PLWH in the ED setting. We therefore based our program design on current recommendations and available evidence from other venues. The major adaptation required to implement these interventions in the ED was the need to link PLWH with appropriate services. We developed this program under the assumption that these services would have beneficial effects as reported in the literature. The efficacy of efforts to link PLWH to services has not been well-reported, and thus we selected linkage rates as a primary process outcome measure for this exploratory evaluation. We contend that implementation of a program for risk reduction counseling and linkage among PLWH in the ED setting addresses the important topic of more closely integrating episodic ED care with the larger health system. This enhances access of PLWH to appropriate services and increases the penetration of case management, medical care and partner notification and referral services among PLWH, such that their previously demonstrated impact on outcome can be maximized for all patients.

### Risk Assessment and Risk Reduction Counseling

Many individuals who learn that they are infected with HIV reduce their risk behavior, yet a significant number continue to engage in behaviors that place others at risk [55-57]. Unprotected sexual behaviors not only place others at risk of infection [8,55], but place PLWH at risk for other sexually transmitted diseases that are co-factors in HIV morbidity and transmissibility [58,59]. Such behaviors have been found to present for between 10% to 60% of patients[4]. One study found that 41% of HIV positive study subjects had unprotected sex, 25% had a new sexually transmitted disease diagnosis, and 15% had used injection drugs since learning of their positive status[6]. Another study showed that 23% of PLWH participating in a clinical trial self-reported unprotected sexual activity during baseline risk-assessment [17]. Among a sample of injection drug users, 66% reported having engaged in HIV-risk behavior since their diagnosis [7]. While these reports have not recruited PLWH from an ED setting, the risk profile among those completing the risk-assessment in our study was similar to prior findings: 25 of 53 patients (47%) for whom data were available admitted to sex in the last six months with a serodiscordant partner, and only 14 (56%) of these stated that they always used condoms.

We have grouped patients by their self-reported ongoing relationship with a medical provider in accordance with the theoretical framework of our program; a primary goal is to link PLWH not actively engaged in healthcare with providers who can address prevention issues on a more sustained basis than is possible in the ED. There is, however, little published evidence to suggest a difference between those in care and those not in care in terms of risky behavior. In one meta-analysis, sexual risk behavior of PLWH was higher among those taking highly active antiretroviral therapy compared to those not in treatment (44% v 33%)[3]. We also found risk behavior to be relatively common among those in care. This, combined with evidence suggesting that patients do not reliably receive recommended prevention measures during clinical encounters[18,19] confirms that the presence or absence of a relationship with a medical provider should not be used as inclusion criteria for our program.

Provider-delivered risk reduction counseling has been shown to reduce risky behavior by PLWH in other settings. A prospective trial of a clinician-delivered intervention, implemented during routine clinical care in two HIV clinics, showed significantly reduced unprotected vaginal and anal intercourse and oral sex over a follow-up interval of 18 months[17]. The CDC recommended PFH intervention was studied in a randomized multi-clinic assessment of HIV provider delivered brief safer-sex counseling[16,39]. Among participants who had two or more sex partners at baseline, unprotected sexual behavior was reduced 38%. It was recognized at implementation that we would likely not be able to replicate these effects as the program was based on a single episode rather than serial encounters. Nonetheless, it was appropriate to include this intervention component for several reasons: 1) to determine the extent to which it is possible for the ED to incorporate current recommendations for prevention intervention in healthcare settings, 2) to create rapport and provide an anchoring for the intervention that patients would see as individualized and acceptable, 3) to engender patient interest in their own health and in HIV prevention, 4) to provide a foundation for subsequent efforts by other providers, and 5) to ethically address problems identified as part of the risk assessment which was required to optimally target linkage efforts.

### Linkage to Interventions

There have been prior studies relevant to the linkage of ED patients to subsequent care. In general there are significant barriers to outpatient follow-up for many ED patients, and establishing linkage is quite challenging[60]. In one study, only 34% of patients without a source of primary medical care successfully followed-up with a medical care center when referred by the ED after being seen for a non-urgent complaint [61]. ED referral for pneumococcal vaccination was successful in 4.9% of those eligible and 9% of those accepting referral[62]. Referral for HIV testing was similarly unsuccessful, even with the addition of patient incentives[63].

Rates of follow-up to a medical provider are significantly improved when a specific appointment is secured for the patient[61,64,65]. A discussion between emergency physicians and on-call specialists, even without a specific appointment time being established, has also been shown to increase rates of patient follow-up from 59% to 79% [65]. The determinants of linkage success for HIV prevention will be unique, but the importance of removing the burden for scheduling and communication from the patient is likely to be broadly applicable. While we attempted to solidify our collaborative relationships, and make our linkage mechanisms systematic and flexible, further work to maximize these linkages is necessary. Strategies to improve linkage might include providing the patient with an appointment time, transportation assistance, written and phone reminders of the appointment, and contacting the patient directly to ensure that the contact occurred. The inability to obtain appointments during non-business hours when many patients present to the ED is a notable barrier we attempted to overcome through mediating linkage. Alternatives might include out-of-hours scheduling through, for example, use of internet-enabled scheduling software.

### Linkage to Healthcare

An effective patient-provider relationship is of primary importance for HIV prevention, as those who receive appropriate treatment are less infectious[66]. Identification and treatment of other sexually transmitted diseases acquired by PLWH also reduces transmission risk [59,67-69]. Linkage with a medical provider has the potential to lead to more general and serial intervention by a medical system that should be increasingly focused on prevention intervention[11]. There has been extensive focus on the importance of linkage between patient and provider at time of diagnosis[37,42,50], but mechanisms to establish or re-establish this relationship for persons already known to have HIV are not well documented. In addition to medical screening and treatment, the CDC has specifically recommended integration of HIV prevention interventions into the clinical care of HIV-infected patients[39]. Clinicians have been called on to affect transmission risk from PLWH by performing screening for high-risk behaviors, communicating and serially reinforcing prevention messages, referring patients for more intensive services such as substance abuse treatment, and facilitating partner counseling and referral services[14]. In our pilot, exploratory evaluation, we were able to facilitate 11 new or renewed medical care relationships, representing 73% of those with an identified need. Re-establishment of care may also diminish morbidity associated with HIV disease progression and lessen the risk of acquired drug resistance. These potential benefits may provide cost reduction that can help finance such programs in the future.

### Linkage to Case Management Services

Case management services are an aggressively recommended component of prevention strategies for PLWH [37]. PLWH are often struggling with unrelated psychosocial factors such as mental illness, substance abuse, and homelessness[37,40-42], and case management can facilitate screening and referral to address these barriers to effective prevention. Case managers can also actively link PLWH to primary care, and help them to overcome barriers to supportive services such as transportation and childcare [37,42,70]. We found that 43% of PLWH felt the need for case management services, although successful linkage was attained in only 4/23 (17%) cases. When the case management agency was contacted by program personnel or patients, appointments were most often unavailable. This is despite collaborative agreements to the contrary prior to program implementation. Collaborating agencies must have the resources necessary to handle an increased number of referrals for coordinated intervention, or efforts by programs such as ours will not result in desired outcomes.

### Linkage to Partner Counseling and Referral Services

Effective partner counseling and referral services are critical to realize the public health benefits of increased screening. Patients find it more difficult than expected to accomplish partner notification on their own[71], but frequently want assistance [72]. Unfortunately, many if not most persons diagnosed with HIV are not interviewed to facilitate partner notification[73,74]. A lack of self-disclosure of HIV status prior to exposure of new partners subsequent to the initial HIV diagnosis is also a recognized and significant problem[56,75-78]. Given the relative deficiency in partner notification for newly diagnosed patients when the importance of partner notification is most emphasized [37,50], it is not surprising that mechanisms for serial assessment of PLWH to facilitate notification of any new partners are not well elucidated. Medical providers are encouraged to assist with partner notification, but success has been historically limited[79]. Therefore, in addition to encouraging patients to disclose HIV status prior to any potential exposure, it is logical to recommend that partner notification should be an ongoing feature of prevention efforts for PLWH who have newly exposed partners.

We found that patients were generally unwilling to disclose partner exposures and did not want referral for partner notification services. Most PLWH who were sexually active (75%) reported that their partner or partners were already aware of their HIV status, and partner notification services were not necessary. However, there were some patients who seemed willing to participate in partner notification and referral services but did not know the names of their past partners and so declined this service. Among the four patients seeking this service, we successfully referred one for partner notification.

### Use of the ED to Provide Prevention Interventions for PLWH

Historically, the focus of discussion for EDs on HIV prevention has been limited to the diagnosis of persons who are unaware of their HIV positive status[80]. We have demonstrated that the ED can have a broader role by addressing the prevention needs, both directly and indirectly, of those who self report their positive HIV status during the ED encounter. We suggest that an ED based intervention should incorporate four basic elements: 1) risk assessment and behavioral counseling, 2) linkage to an ongoing relationship with a medical provider, 3) linkage to social support and case management services, and 4) linkage to partner referral and counseling services.

The implementation of ED prevention programs must be balanced with resource availability and interest in public health intervention. Despite slowly accelerating progress, prevention interventions in the ED have been historically limited and remain controversial. Resistance to their implementation has yet to undergo rigorous study, perhaps because the reasons seem intuitively obvious. First, emergency care providers are necessarily focused on assessment and stabilization of patients with a medical emergency. The core element of that evaluation is deciding what has to be done immediately and what can wait. The second is that EDs are sufficiently overwhelmed to reject any additional activity as a burden to existing patients and providers [81-83]. It follows that additional services should require either additional resources or a justification of what other service can be delayed or eliminated to enable the new activity.

Given the controversial nature of offering prevention activities within the ED setting, one might question whether a similar service could be accomplished in an alternative setting. Referrals from prisons, substance abuse treatment centers or other venues that frequently care for disadvantaged patients are one likely possibility. Patients might also be recruited through outreach, though this is likely to be more difficult than passively receiving patients in a setting where an opportunity for interaction exists. General increase in service availability might have some effect; perhaps some patients have tried at one point to access services but felt them to be unavailable. Peer recruitment might also be considered as it has been to increase HIV testing [84]. In one report, women who used drugs and had acquired or were at risk for HIV infection were recruited through street outreach, needle exchange sites, a prison, and local community based organizations to study the service needs of out-of-treatment drug users and the ability of an interactive case management intervention to address those needs. Case management was most successful in meeting needs for supportive mental health counseling, basic services, and long term housing but was less effective for accessing medical and dental services[85].

There is little data to guide conjecture on the relative costs of providing a program such as ours in the ED as opposed to another venue. This program received $39,320 annually to support an addition to infrastructure that was already in place. This equates to approximately $714 per patient given current rates of enrollment. We do not know how this translates to cost-effectiveness. For comparison, our previously reported screening program cost per newly identified patient in this relatively low prevalence area was $3,113 [36] The annual cost of an AIDS patient with highly active anti-retroviral therapy has been estimated to be $10,998[86]. Given these points of reference, we would suggest that even a small benefit of our intervention might justify the required expenditure. However, the cost to implement such a program without a pre-existing counselor infrastructure is difficult to estimate.

We have not systematically assessed provider acceptance of our program. Our program is somewhat unique in that it endorses the notion that little will be done by existing ED providers to provide for the prevention needs of PLWH, but it also recognizes that the ED can serve as a setting in which to identify those in need of prevention services and facilitate linkage. Since referral of patients to resources outside the ED for non-emergent needs is a central tenet of emergency practice, this program should be conceptually compatible to emergency physicians if resources are adequate. A level of disinterest in the program was suggested by the limited referral of PLWH to counselors by medical staff, but anecdote suggests that providers seemed largely neutral or unaware of the program's activity rather than being frankly unsupportive. This argues in favor of feasibility if sufficient resources are provided to avoid detraction from the primary emergency medicine mission.

## Limitations

While our data demonstrate that it is feasible to implement a program designed to address the prevention needs of PLWH in an ED setting, several caveats are necessary to appropriately interpret our results. Most notably, the resources for such intervention are not readily available in most EDs; our program's implementation was facilitated by the counseling and testing program already in place. We have defined program effectiveness in terms of successful linkage of underserved patients to existing resources, but it is unknown whether patients would have self-referred for services in the absence of the linkage program. We consider successful linkage as a valid primary outcome for this exploratory evaluation, because 1) referrals are a core activity of emergency medical providers for non-emergent complaints, and 2) medical care, case management, and partner notification and referral services are of proven benefit. However, evaluation of patient outcomes would be necessary before firm recommendations for broad translation of our program can be made. Evaluation of barriers to implementation would also be of benefit. This should include assessment of the impact of the intervention on ED length-of-stay, any effects on aggregate ED service delivery, and ED staff perceptions and willingness to support such a program.

We did not include those patients who refused or were not approached in our calculations of intervention effectiveness. The total number of eligible patients is unknown; while billing data provides a gross indication of the number of patients with HIV recorded in the medical record, the eligibility of these patients for participation is unclear. The effectiveness of the program would be reduced by the degree of failure in accessing every eligible patient presenting to the ED. Also, risk assessment was not performed for refusing patients. If these patients had significant levels of unmet need or high levels of ongoing risky behavior then our intervention would be less effective than we have reported.

Despite these limitations, we suggest that the implementation of our program, and the success of just a few referrals or partner notifications, represents a significant advancement in prevention interventions for PLWH by accessing traditionally underserved patients and more closely linking the ED with the broader health system.

## Conclusion

Our experience demonstrates that, given the resources of trained counselors, it is feasible to implement a program of prevention interventions for persons living with HIV in the emergency department. Emergency department patients living with HIV often have unmet needs for clinical and prevention services. Due to the difficulties inherent in accomplishing prevention goals within the overburdened system of emergency care, we contend that ED based prevention for PLWH should focus primarily on linkage to those community resources where validated and ongoing interventions can feasibly be delivered. Nonetheless, it might be possible to attenuate healthcare and prevention barriers for PLWH if emergency departments incorporated CDC recommended prevention measures for healthcare providers.

## Competing interests

The author(s) declare that they have no competing interests.

## Authors' contributions

MSL, DLR, CJL, ATT, and CJF participated in the conception and design of the program and study. MSL and DLR obtained IRB approval. DLR acquired the data. CJL provided statistical advice and data were primarily analyzed by DLR. CJF, ATT, and CJL assisted MSL and DLR in the supervision of the clinical program from which the data were collected. MSL, DLR, CJL drafted the manuscript and all authors contributed substantially to its revision. MSL takes responsibility for the paper as a whole. All authors have read and approved the manuscript.

**Figure 1:**
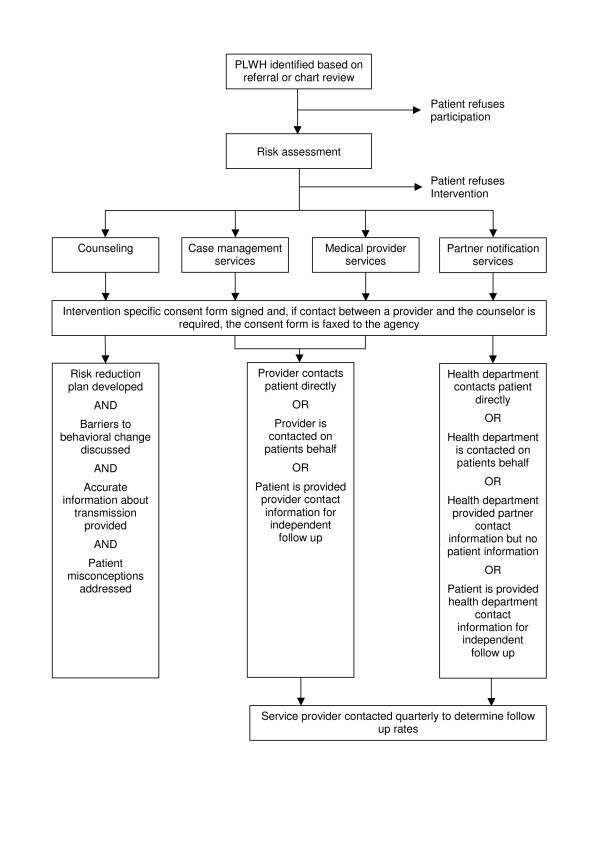
Prevention for Positives Intervention Process.

## Pre-publication history

The pre-publication history for this paper can be accessed here:


